# Supplementary material for: Building electrode skins for ultra-stable potassium metal batteries
Source: Nat Commun. 2023 Apr 21;14:2305. doi: 10.1038/s41467-023-38065-9 (PMC10121571; doi:10.1038/s41467-023-38065-9)
Supplement: Supplementary file 1 — Supplementary Information [file 41467_2023_38065_MOESM1_ESM.pdf]

Supplementary Information

Building electrode skins for ultra-stable potassium metal batteries

Hongbo Ding[1], Jue Wang[2], Jiang Zhou[3], Chengxin Wang[4,*] and Bingan Lu[1,*]

[1]School of Physics and Electronics, State Key Laboratory of Advanced Design and Manufacturing for Vehicle Body, Hunan University, Changsha 410082, China.

[2]College of Chemistry and Chemical Engineering, Central South University, Changsha 410083, China.

[3]School of Materials Science and Engineering and Key Laboratory of Nonferrous Metal Materials Science and Engineering, Ministry of Education, Central South University, Changsha 410083, China.

[4]State Key Laboratory of Optoelectronic Materials and Technologies, School of Materials Science and Engineering, Sun Yat-sen (Zhongshan) University, Guangzhou, 510275, China.

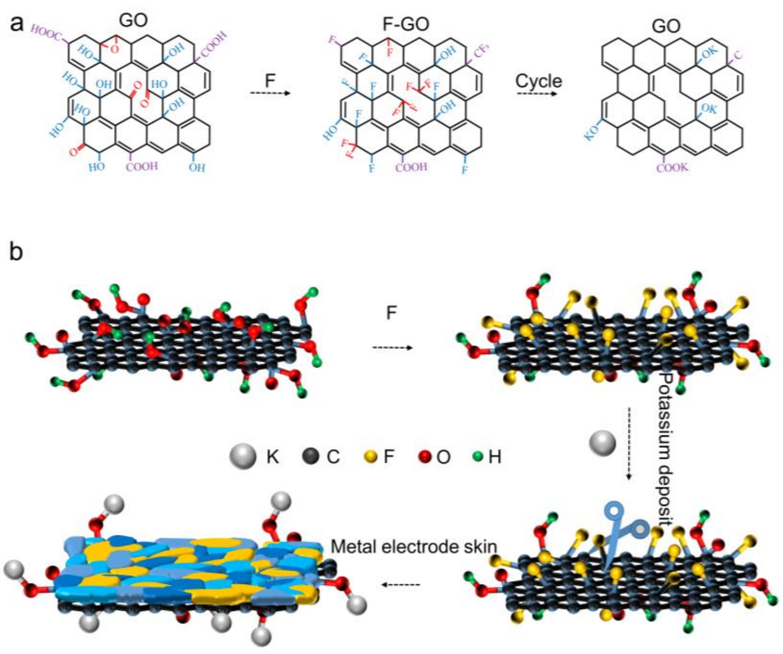

Supplementary Figure 1. Synthesis F-GO and MES. (a, b) Synthesis of F-GO and evolution of metal electrode skins.

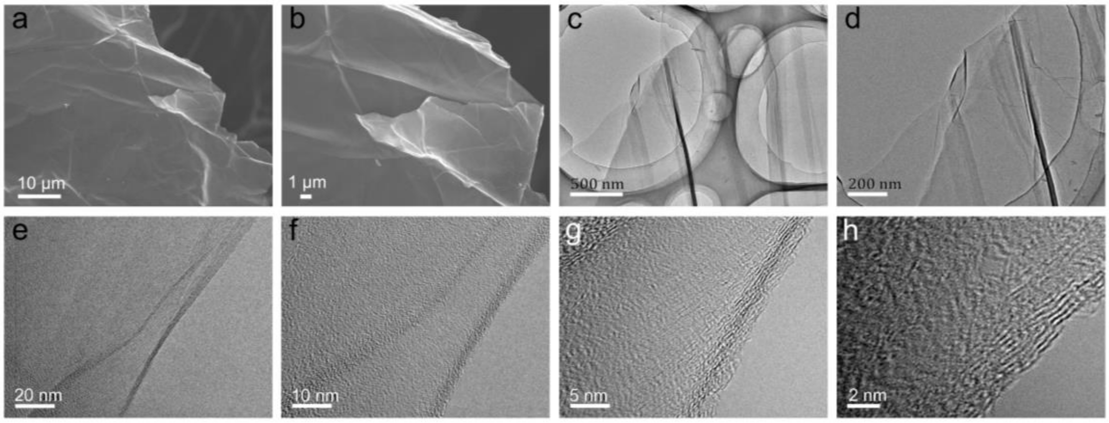

Supplementary Figure 2. Morphology of GO. SEM (a, b) and TEM (c-h) image of GO.

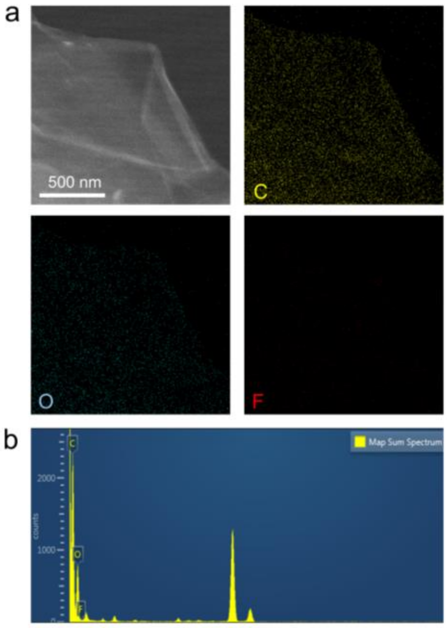

Supplementary Figure 3. Elements Maps of GO. EDS maps (a) and element content (b) of the GO.

Name	Peak KE	FWHM eV	Area (P) CPS.eV	Atomic %
C	285.81	4.48	196076.42	66.56
O	532.06	3.34	237051.37	31.52
S	167.95	4.32	5925.19	1.14
Total:				99.22

Table 1. Elements content of GO. The content and proportion of carbon and oxygen in GO.

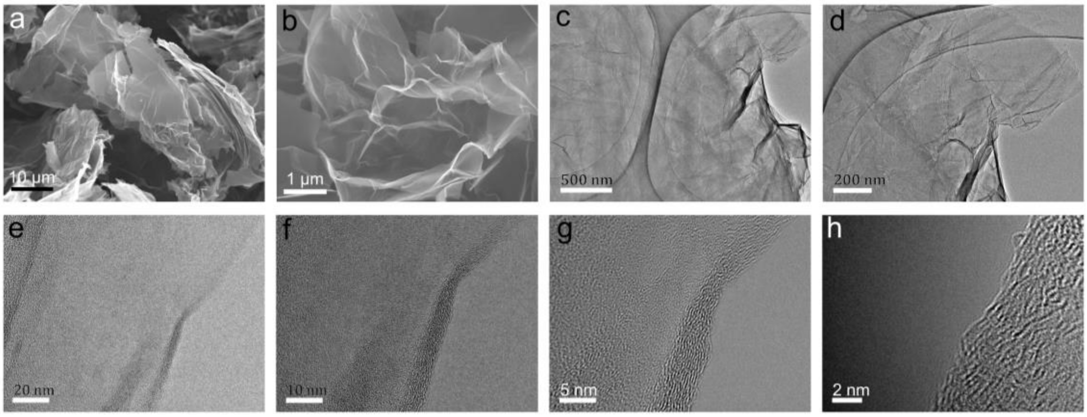

Supplementary Figure 4. Morphology of F-GO. SEM (a, b) and TEM (c-h) image of F-GO.

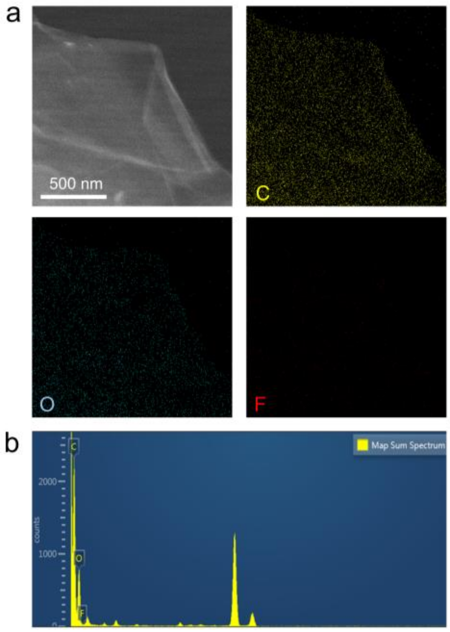

Supplementary Figure 5. Elements Maps of F-GO. EDS maps (a) and element content (b) of the F-GO.

Name	Peak KE	FWHM eV	Area (P) CPS.eV	Atomic %
C	285.81	4.48	196076.42	66.56
O	532.06	3.34	237051.37	31.52
S	167.95	4.32	5925.19	1.14
Total:				99.22

Table 2. Elements content of F-GO. The content and proportion of carbon and oxygen in F-GO.

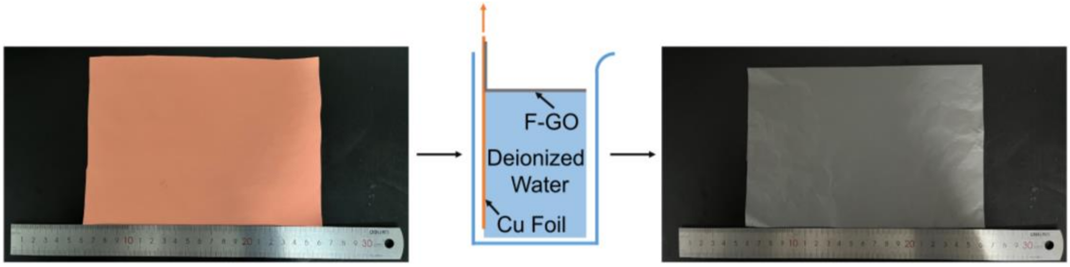

Supplementary Figure 6. Digital photos of Cu and Cu@F-GO. Preparation of Cu@F-GO.

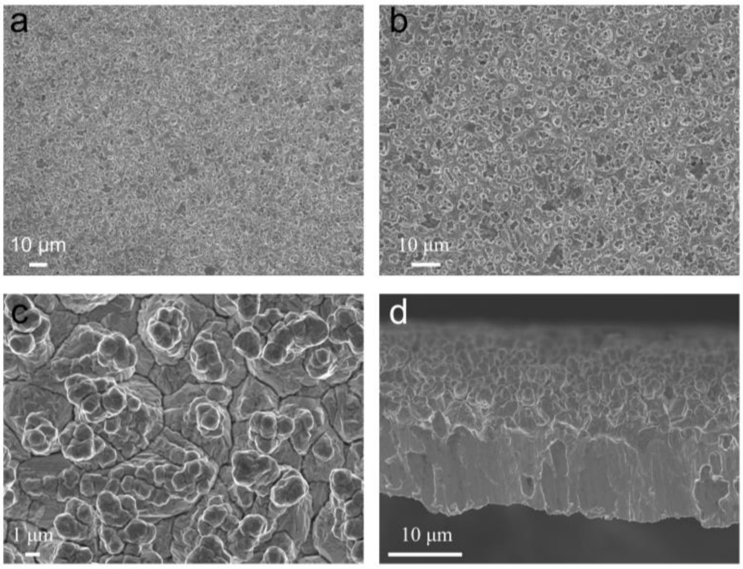

Supplementary Figure 7. SEM image of the Cu foil. Surface (a-c) and cross-section (d) of Cu.

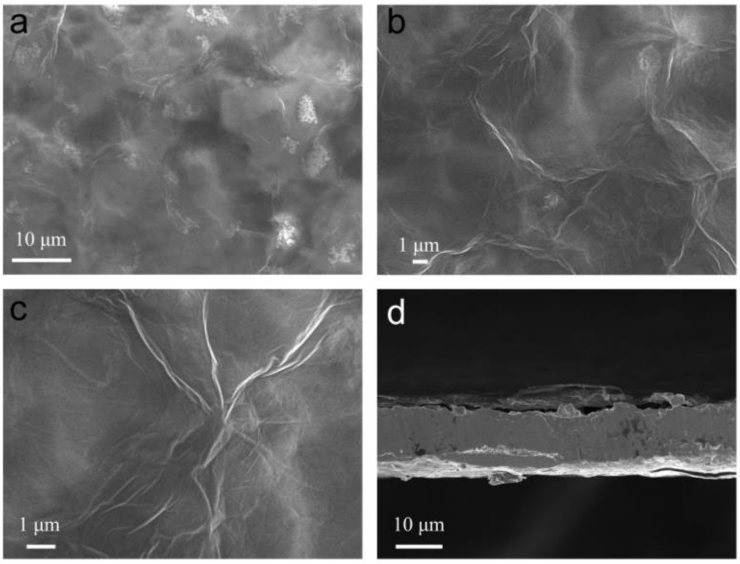

Supplementary Figure 8. SEM image of the Cu@F-GO. Surface (a-c) and cross-section (d) of Cu@F-GO.

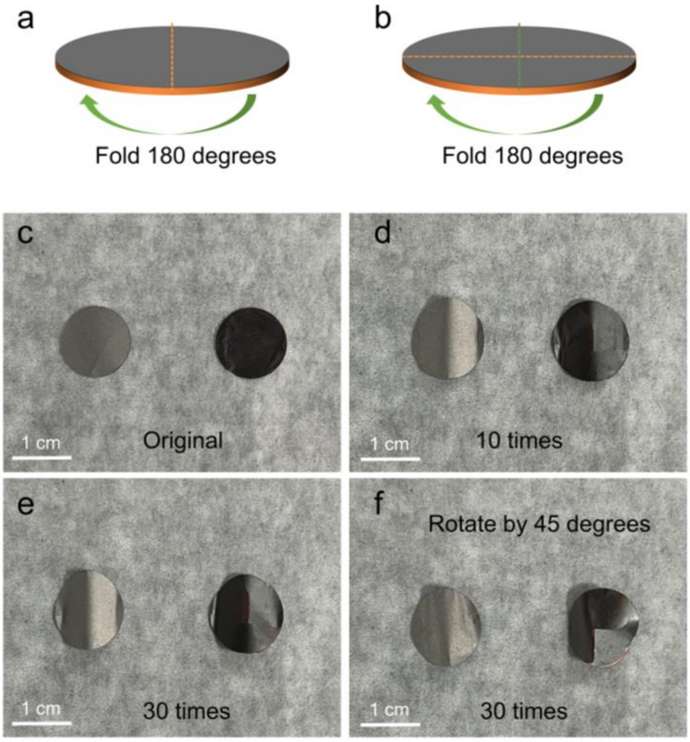

Supplementary Figure 9. Metal fatigue test for MES and GO. (a, b) Folding mode of metal fatigue experiment. (c) Cu@MES and Cu@GO in their original state. (d) Cu@MES and Cu@GO fold ten times (according to the method in a). (e) Cu@MES and Cu@GO fold thirty times (according to the method in Figure a). (f) Cu@MES and Cu@GO fold thirty times (according to the method in b).

Supplementary Fig. 9a and 9b show two schematic diagrams of folding in different directions. The metal fatigue properties of F-GO and GO are verified by folding tests on Cu@F-GO and Cu@GO. In the original state, both polar plates showed good integrity (Supplementary Fig. 9c). First of all, the folding experiment is carried out according to the first folding mode. After ten folds, F-GO still maintains structural stability, while cracks appear on GO surface (Supplementary Fig. 9d). After 30 folds, GO breaks but F-GO remains structurally stable (Supplementary Fig. 9e). By using another folding method for 30 times, the structure of F-GO did not change, but GO had

a wide range of cracks (Supplementary Fig. 9f). These results indicate that F-GO has better metal fatigue performance.

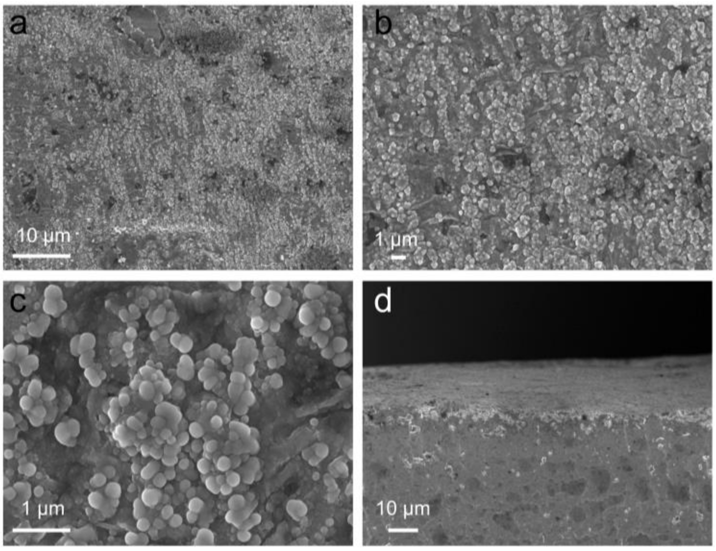

Supplementary Figure 10. SEM image of the bare K. Surface (a-c) and cross-section (d) of bare K.

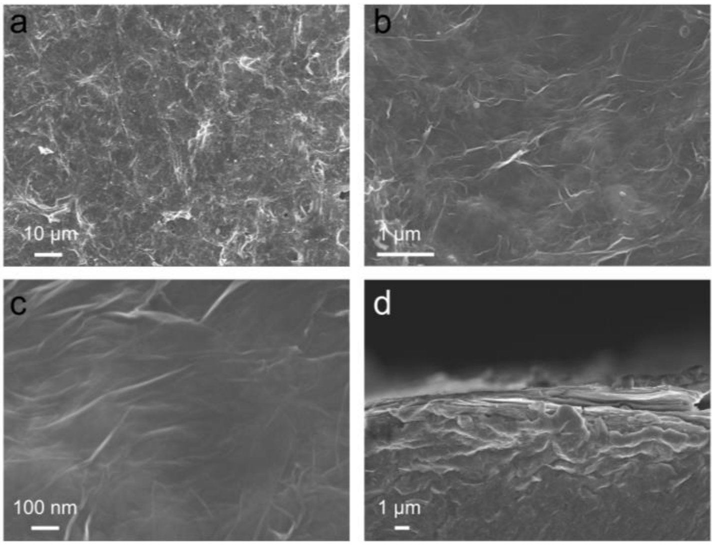

Supplementary Figure 11. SEM image of the K@F-GO. Surface (a-c) and cross-section (d) of K@F-GO.

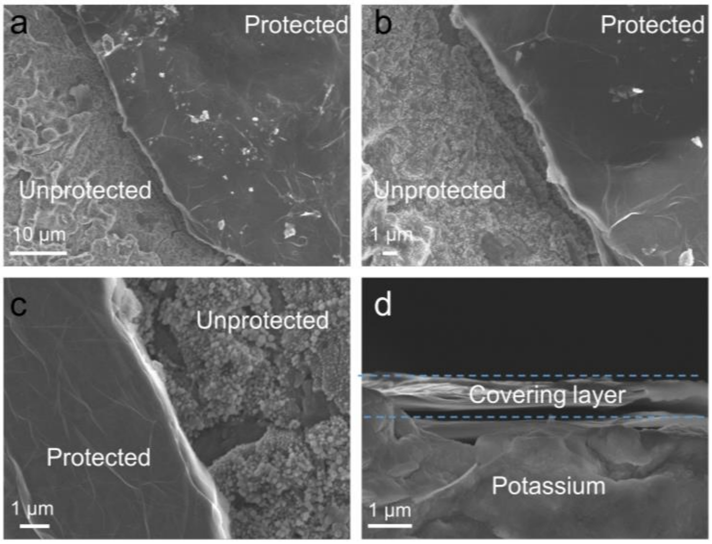

Supplementary Figure 12. SEM image of the K@F-GO and bare K. (a-c) Protected and unprotected potassium metal surfaces. (d) Potassium metal cross section with protective layer.

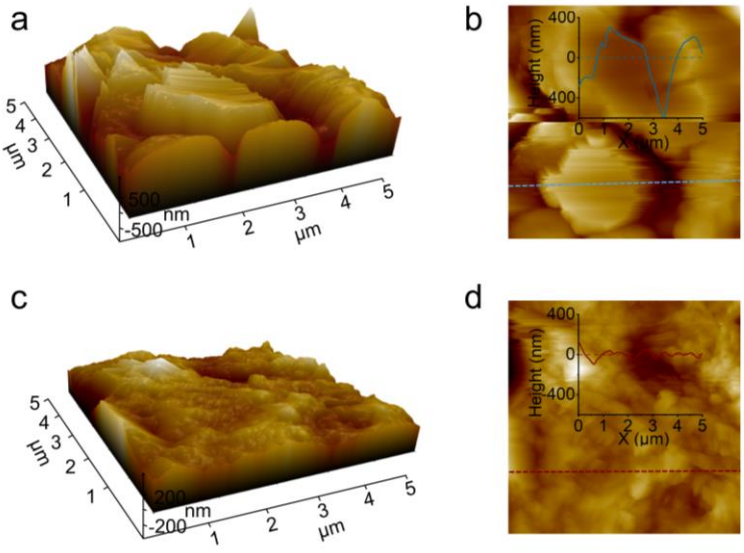

Supplementary Figure 13. Surface morphology of Cu and Cu@MES. AFM images and surface roughness of Cu foil (a, b) and Cu@MES (c, d).

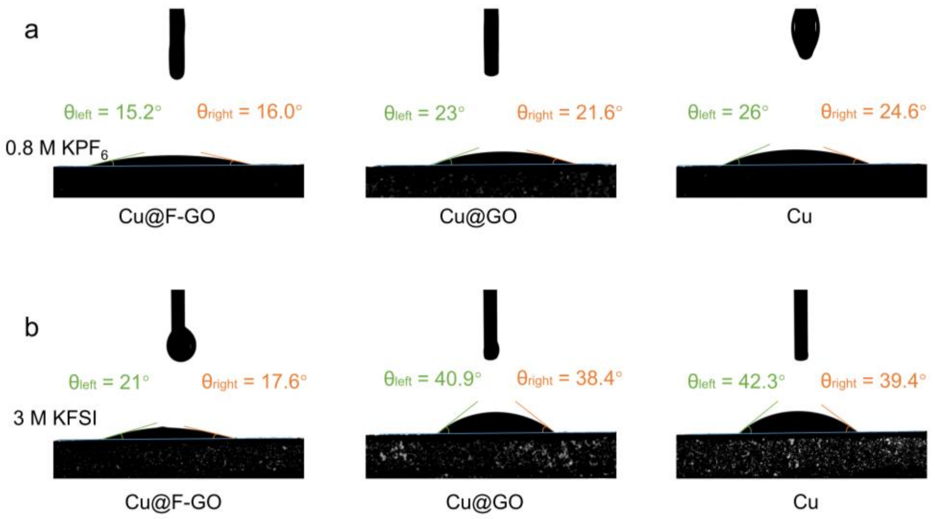

Supplementary Figure 14. Contact angles of three materials in different electrolytes. (a) Contact Angle of Cu@F-GO, Cu@GO and Cu at 0.8 M KPF_6. (b) Contact Angle of Cu@F-GO, Cu@GO and Cu at 3 M KFSI.

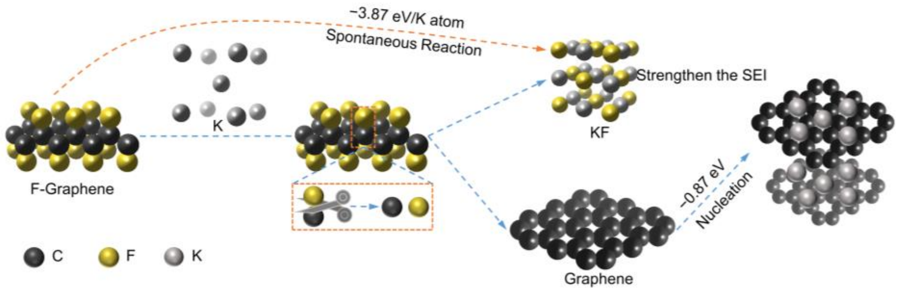

Supplementary Figure 15. Optimized structures of each modeling substrate. In the models, the K, fluorine (F) and carbon (C) atoms are displayed as spheres in silver, yellow, and black, respectively.

Type	K	F-Graphene	KF	Graphene
Lattice Parameters	5.26605 Å	2.57726 Å	3.80230 Å	2.46759 Å
ΔG	K + F-Graphene ⟶ KF + Graphene −3.87 eV/K atom			

Table 3. Lattice parameters of each modeling substrate. The lattice parameter of K, graphene fluoride, potassium fluoride (KF) and graphene.

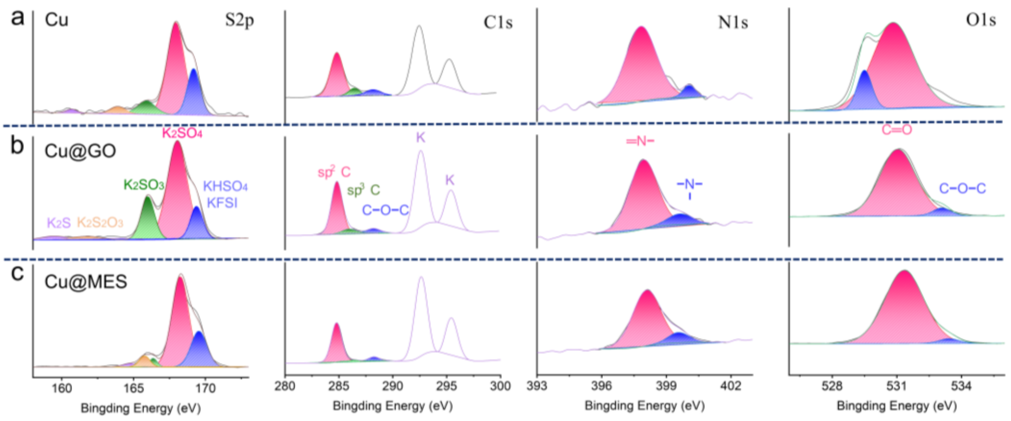

Supplementary Figure 16. High-resolution of XPS spectra S2p, C1s, N1s and O1s XPS curves after 10 cycles with 3 M KFSI in DME as electrolyte. Cu (a), Cu@GO (b) and Cu@MES (c).

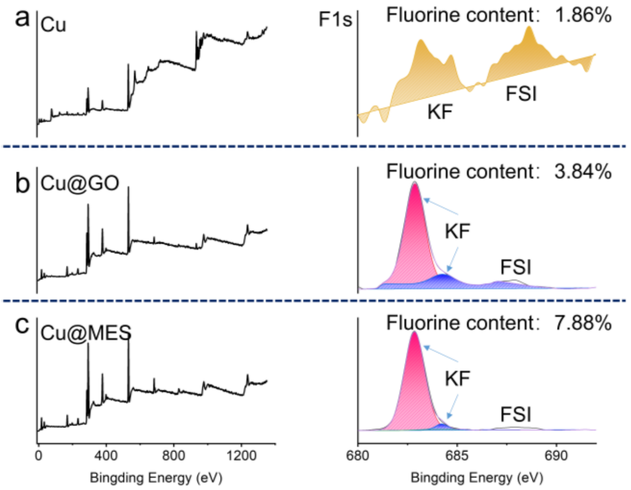

Supplementary Figure 17. Full survey XPS spectra, high resolution F1s XPS spectra and fluoride content after 10 cycles with 3 M KFSI in DME as electrolyte. Cu (a), Cu@GO (b) and Cu@MES (c).

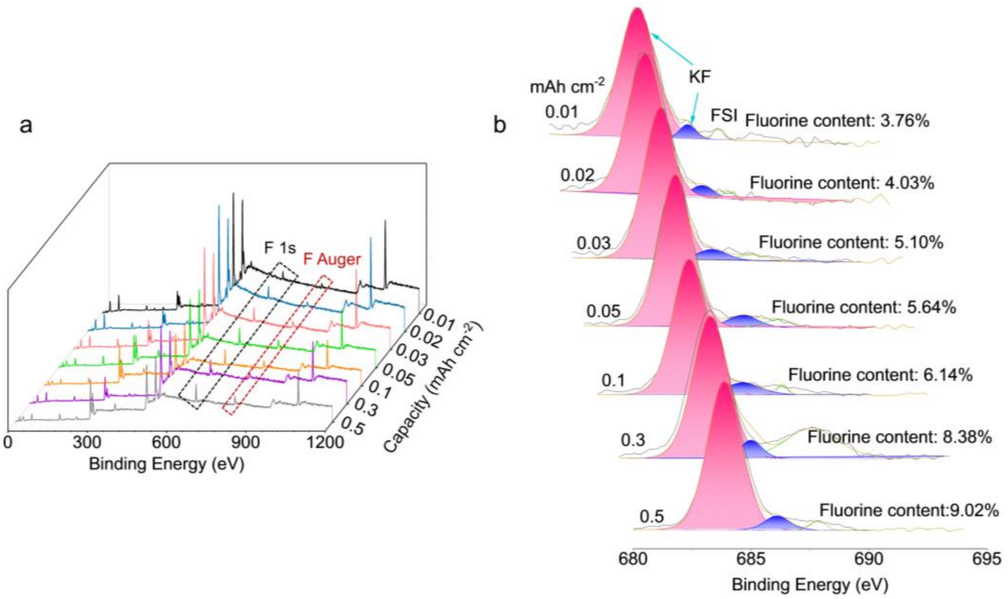

Supplementary Figure 18. The XPS spectra of MES after different area capacity with 3 M KFSI in DME as electrolyte. (a) Full survey XPS spectra. (b) High resolution F1s XPS spectra and fluoride content.

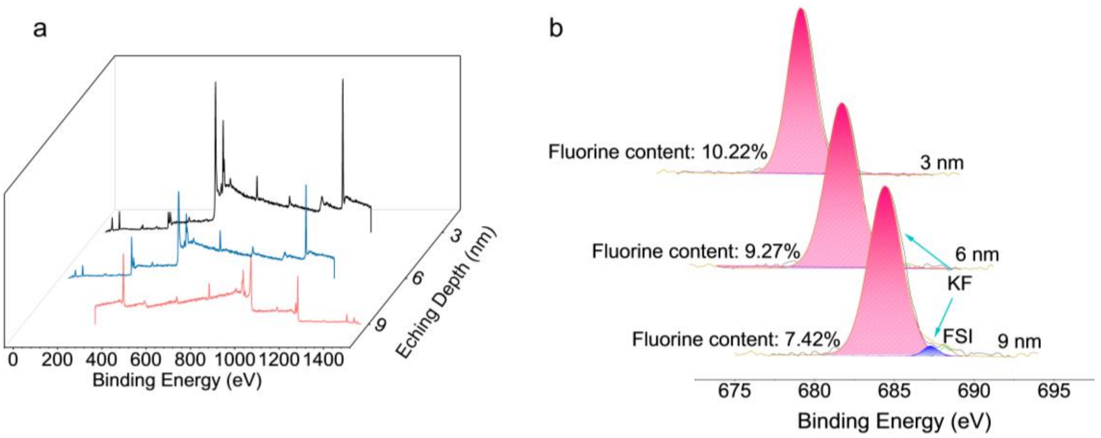

Supplementary Figure 19. The XPS spectra of MES after different depth of etching with 3 M KFSI in DME as electrolyte (0.5mAh cm^{-2}). (a) Full survey XPS spectra. (b) High resolution F1s XPS spectra and fluoride content.

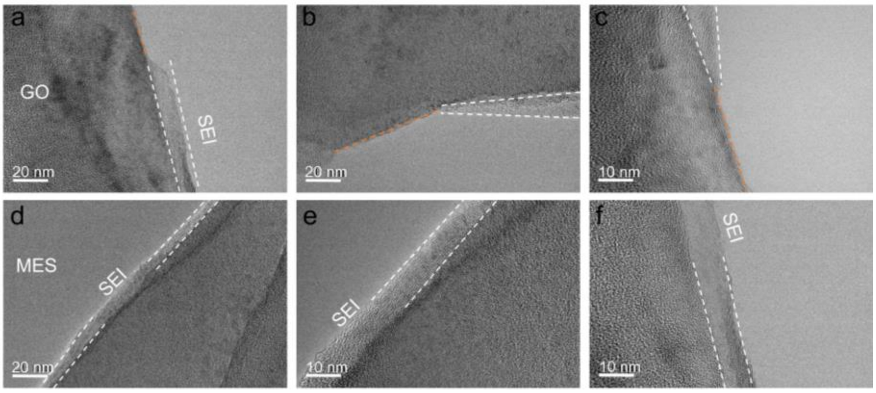

Supplementary Figure 20. TEM and HRTEM image. The SEI in GO (a-c) and MES (d-f) after 10 cycles with 3 M KFSI in DME as electrolyte.

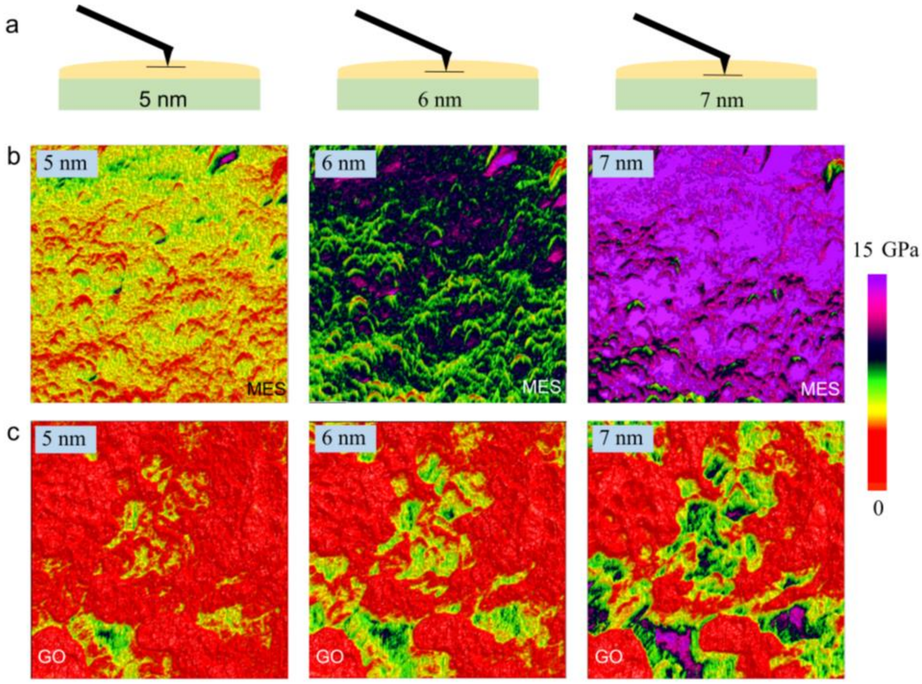

Supplementary Figure 21. SEI modulus for F-GO and GO surface after 50 cycles. (a) Schematic of the AFM tapping mode to test the modulus of SEI. The tapping depth increases stepwise, from 5, 6 to 7 nm. The 2D modulus mapping of each depth of SEI layers for (b) F-GO and (c) GO respectively.

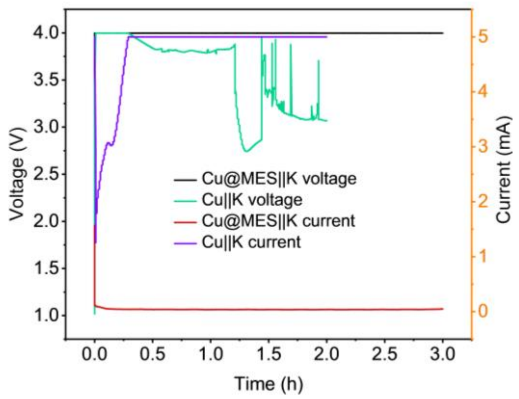

Supplementary Figure 22. Constant voltage test. Potential holding test of the Cu@MES and Cu electrode at 4 V vs. K^+/K (0.8 M KPF_6).

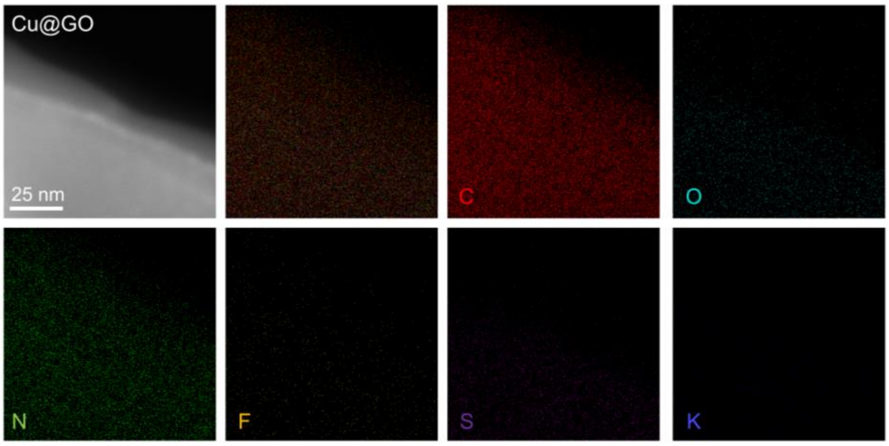

Supplementary Figure 23. SEI element mappings of Cu@GO. The element mappings of SEI in the GO after 10 cycles with 3 M KFSI in DME as electrolyte.

Element	C	O	N	F	S	K
Wt%	91.72	2.69	4.00	0.39	0.12	1.08

Table 4. SEI element content of Cu@GO. The proportion of various elements in the SEI.

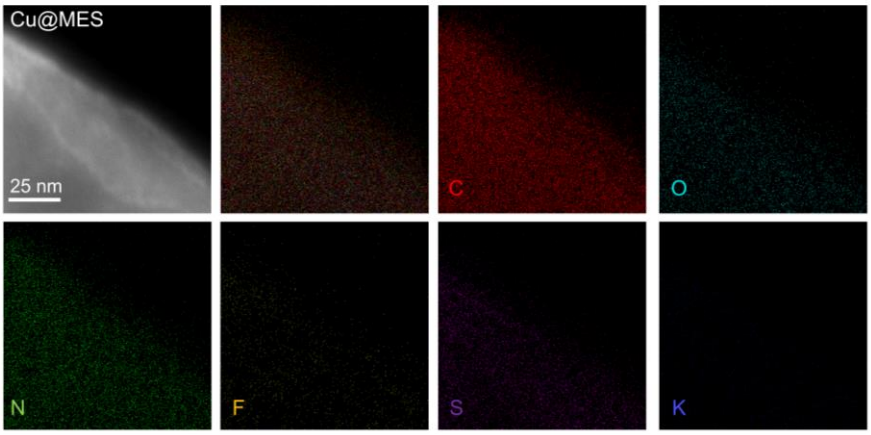

Supplementary Figure 24. SEI element mappings of Cu@MES. The element mappings of SEI in the MES after 10 cycles with 3 M KFSI in DME as electrolyte.

Element	C	O	N	F	S	K
Wt%	86.50	4.91	4.92	0.78	0.24	2.64

Table 5. SEI element content of Cu@MES. The proportion of various elements in the SEI.

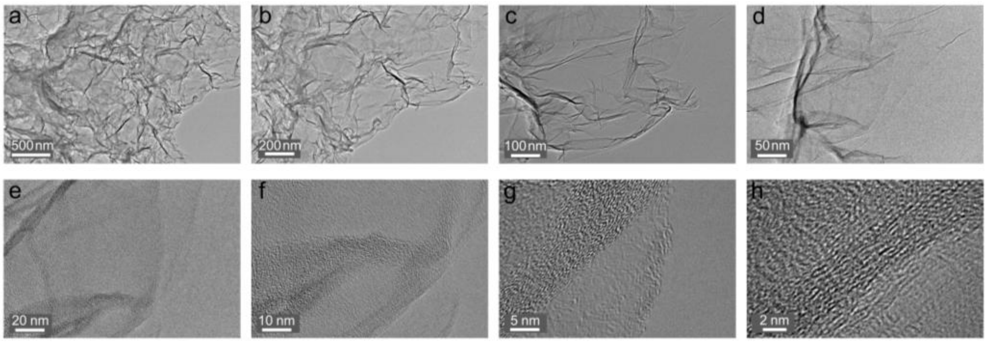

Supplementary Figure 25. TEM image of pure F-GO. (a-h) TEM image of pure F-GO after K metal plating/stripping.

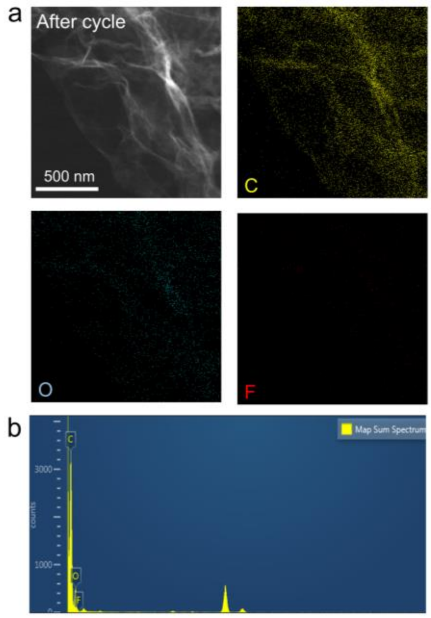

Supplementary Figure 26. Elements Maps and content of F-GO after cycles. EDS maps (a) and element content (b) of the F-GO after cycles.

Name	Peak KE	FWHM eV	Area (P) CPS.eV	Atomic %
C	284.8	3.44	224680.37	85.95
O	529.38	4.67	79796.62	11.96
N	399.75	0.99	2753.30	0.62
Total:				98.53

Table 6. Elements content of F-GO after cycles. Element content of pure F-GO after K metal plating/stripping

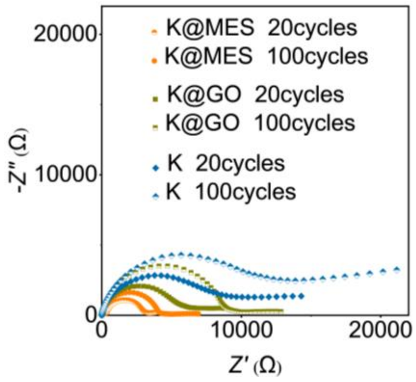

Supplementary Figure 27. Impedance measurement. Impedance of three symmetrical cells at different cycles.

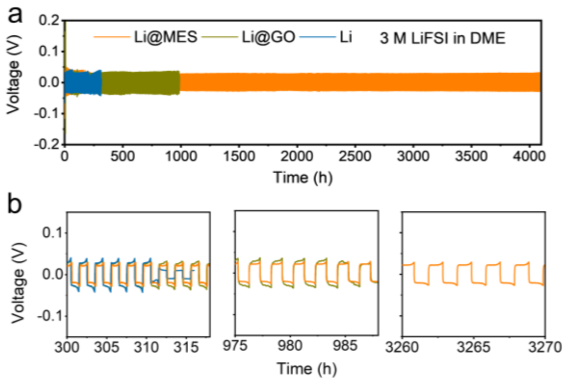

Supplementary Figure 28. Galvanostatic Li plating/stripping voltage profiles for the Li||Li symmetric cells. Current density of 1 mA cm^{-2} and capacity of 1 mAh cm^{-2} with LiFSI electrolyte.

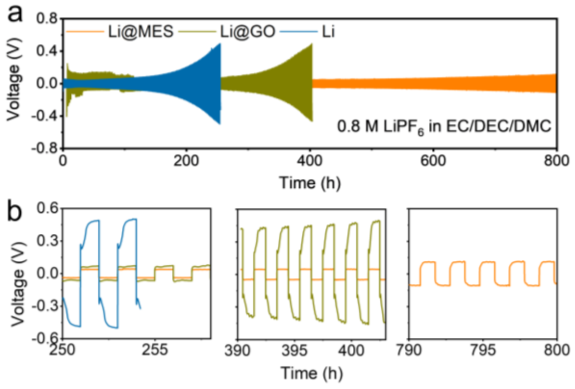

Supplementary Figure 29. Galvanostatic Li plating/stripping voltage profiles for the Li||Li symmetric cells. Current density of 1 mA cm^{-2} and capacity of 1 mAh cm^{-2} with $LiPF_6$ electrolyte.

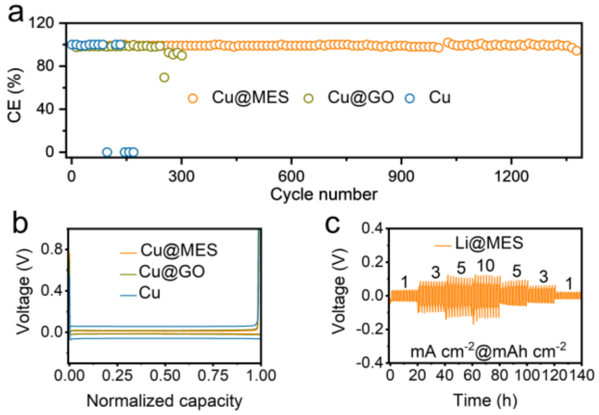

Supplementary Figure 30. Li-Cu asymmetric battery performance. (a) Coulombic

efficiency as a function of cycle number for Cu||K asymmetric cells with LiFSI electrolyte and (b) corresponding voltage profiles. (c) Voltage profiles of symmetric Li||Li cells at various current densities.

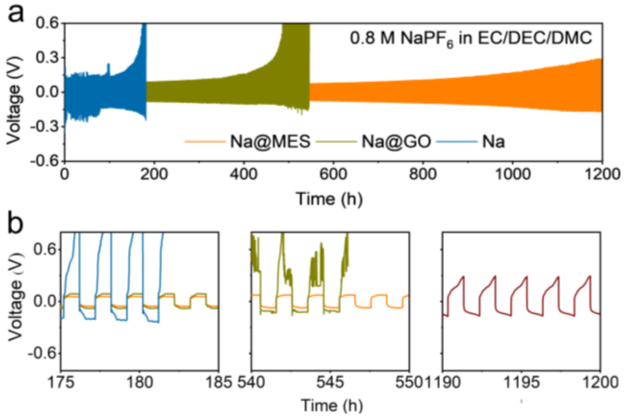

Supplementary Figure 31. Galvanostatic Na plating/stripping voltage profiles for the Na||Na symmetric cells. Current density of 0.1 mA cm^{-2} and capacity of 0.1 mAh cm^{-2} with $NaPF_6$ electrolyte.

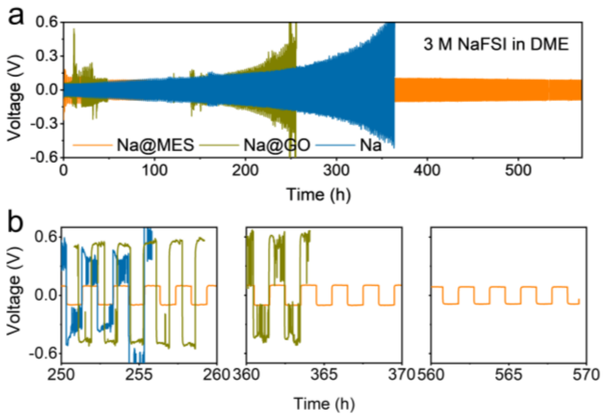

Supplementary Figure 32. Galvanostatic Na plating/stripping voltage profiles for the Na||Na symmetric cells. Current density of 0.1 mA cm^{-2} and capacity of 0.1 mAh cm^{-2} with NaFSI electrolyte.

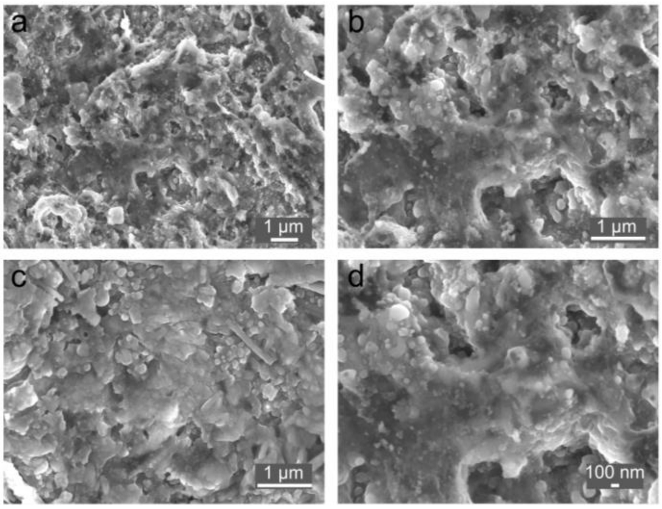

Supplementary Figure 33. Morphology characterization of the plated K. (a-d) SEM image of bare K surface after cycling.

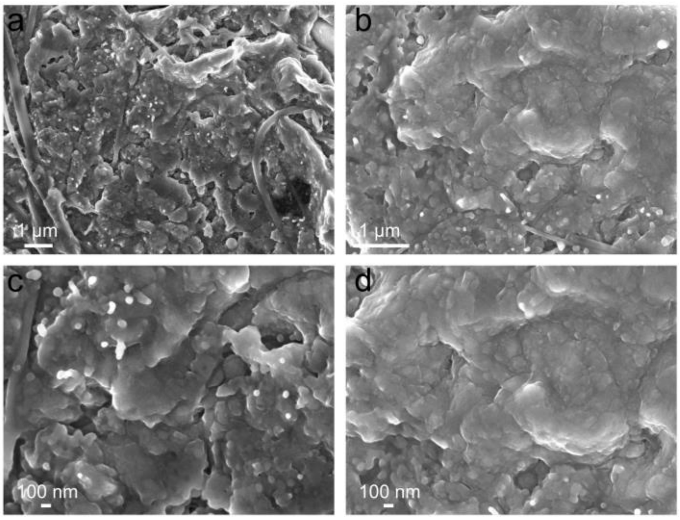

Supplementary Figure 34. Morphology characterization of the plated K@GO. (a-d) SEM image of bare K@GO surface after cycling.

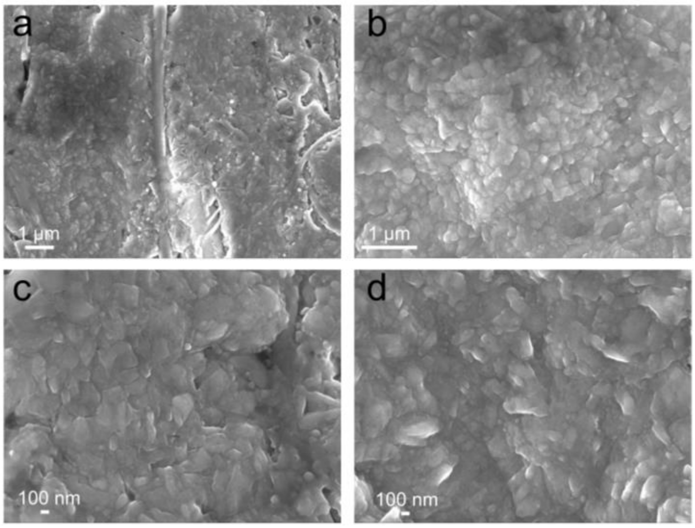

Supplementary Figure 35. Morphology characterization of the plated K@MES.

(a-d) SEM image of bare K@MES surface after cycling.

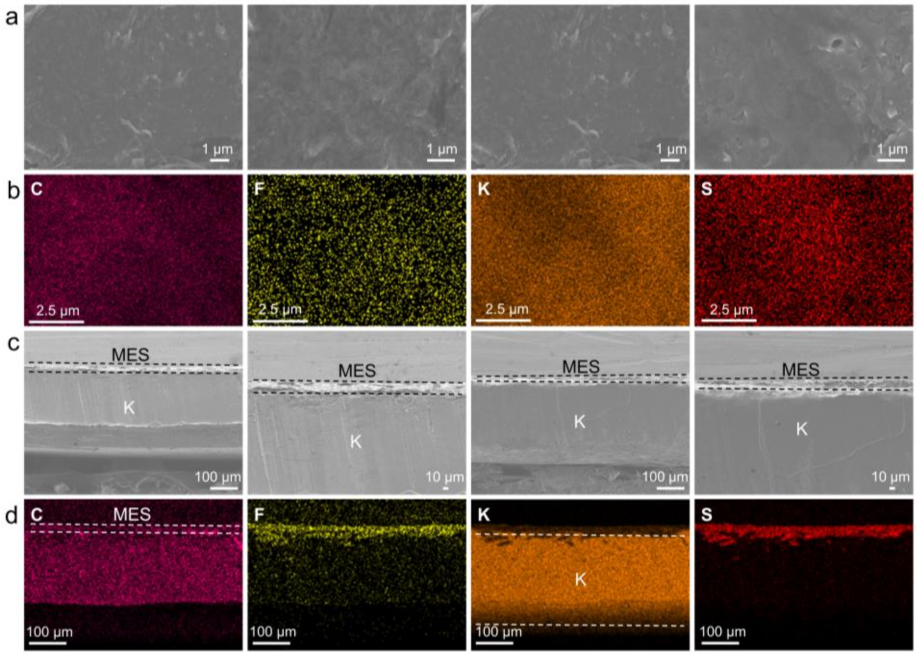

Supplementary Figure 36. Morphology and structure after K@MES stripping 0.5 mAh cm^{-2}. (a, b) SEM image and EDS mapping of surface. (c, d) SEM image and EDS mapping of cross profile.

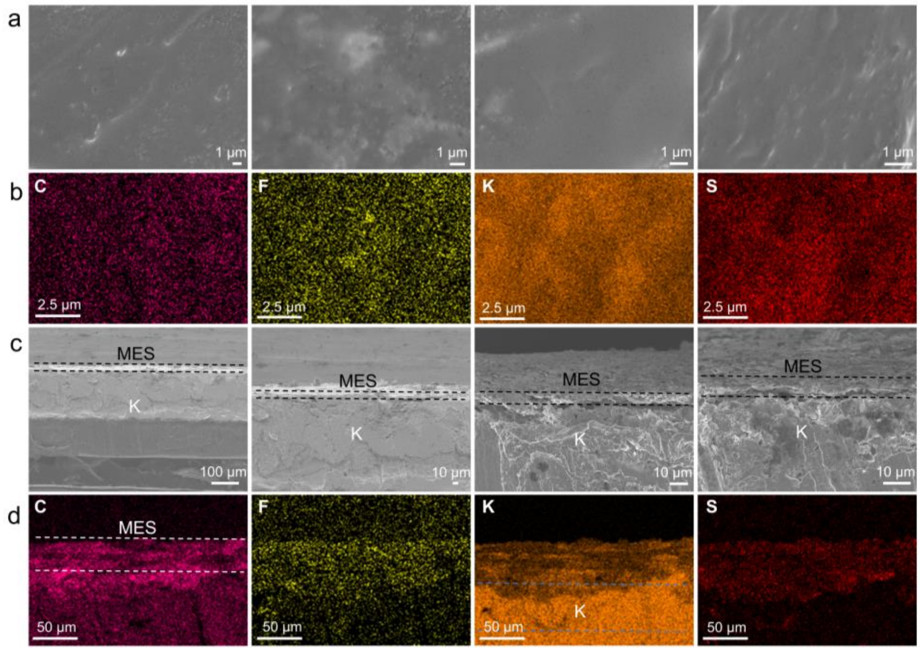

Supplementary Figure 37. Morphology and structure after K@MES plating 0.5 mAh cm^{-2}. (a, b) SEM image and EDS mapping of surface. (c, d) SEM image and EDS mapping of cross profile.

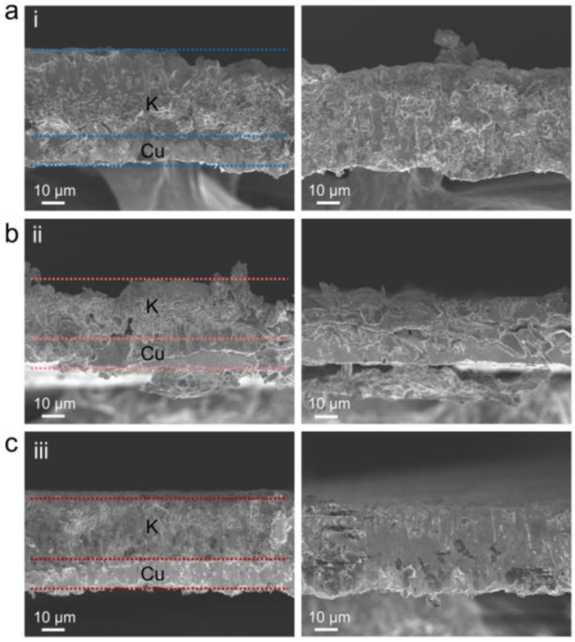

Supplementary Figure 38. SEM cross-sectional images of K metal deposited on different current collectors (0.5 mA cm^{-2}, 2 mAh cm^{-2}). (a) Cu, (b) Cu@GO, (c) Cu@MES.

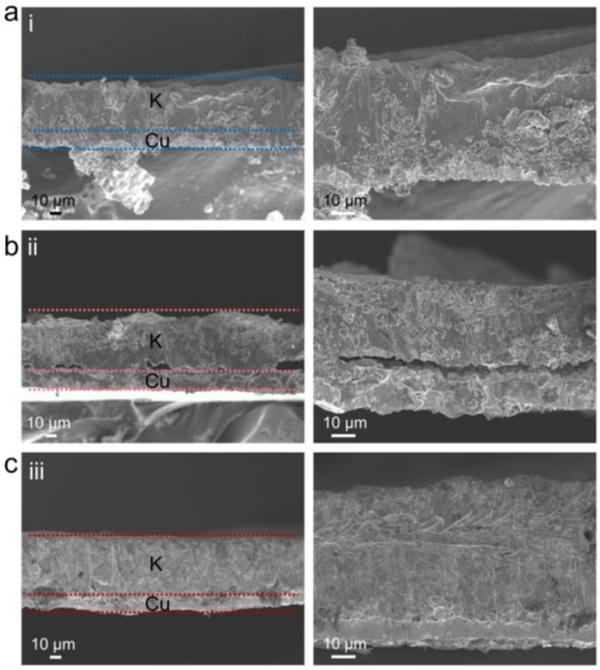

Supplementary Figure 39. SEM cross-sectional images of K metal deposited on different current collectors (0.5 mA cm^{-2}, 5 mAh cm^{-2}). (a) Cu, (b) Cu@GO, (c) Cu@MES.

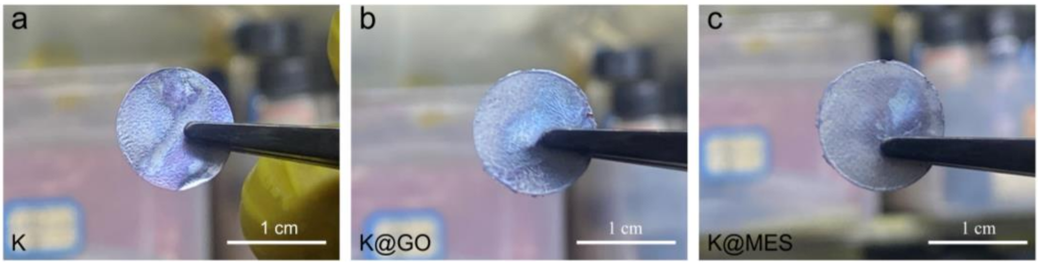

Supplementary Figure 40. Optical photograph of K metal deposited on different current collectors (0.5 mA cm^{-2}, 5 mAh cm^{-2}). (a) Cu, (b) Cu@GO, (c) Cu@MES.

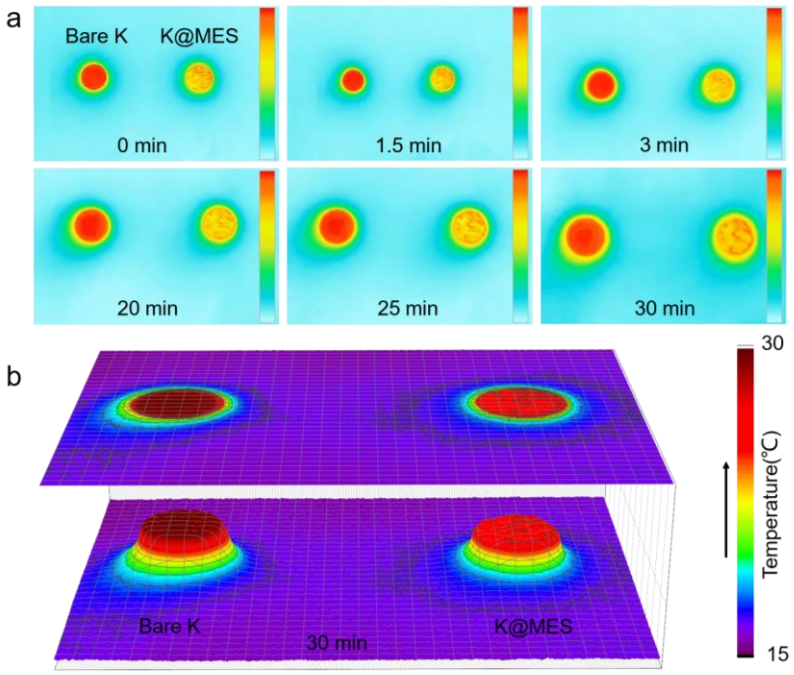

Supplementary Figure 41. Potassium metal exothermic test. (a, b) Exothermic state of Bare K and K@MES after reaction with air.

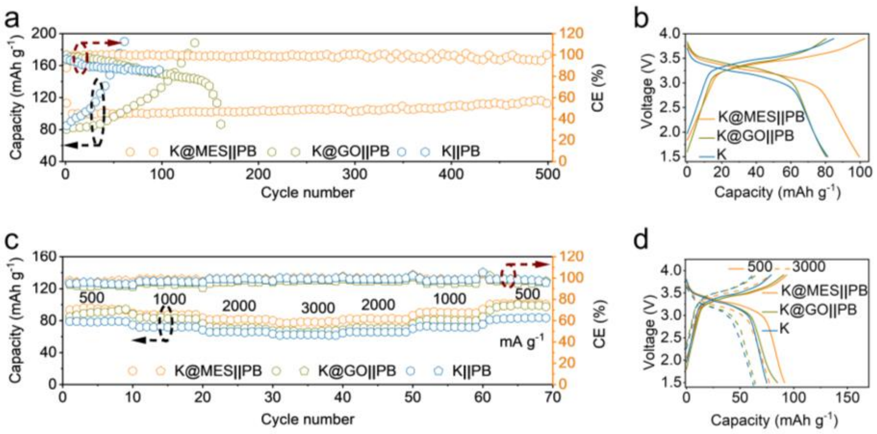

Supplementary Figure 42. Electrochemical performance of K||PB coin cells. (a) Cycling performances and charge and discharge voltage profiles (b) of the K||PB cells at a specific current of 500 mA g^{-1} using KPF_6 electrolytes. Rate performance (c) and the corresponding charge and (d) discharge voltage profiles of the K||PB cells.
